# Supplementary material for: Patterns of Intron Gain and Loss in Fungi
Source: PLoS Biol. 2004 Nov 30;2(12):e422. doi: 10.1371/journal.pbio.0020422 (PMC532390; doi:10.1371/journal.pbio.0020422)
Supplement: Table S1 — Also available at http://genes.mit.edu/NielsenEtAl/. (4.3 MB ZIP). [file pbio.0020422.st001.zip › NielsenEtAl/html/282.html]

AN7354.1.NCU00464.1.MG05248.1.FG04137.1


```
 CLUSTAL W (1.82) Multiple Sequence Alignments - Introns Inserted


Sequence 1: NCU00464.1	131 aa
Sequence 2: FG04137.1	125 aa
Sequence 3: MG05248.1	135 aa
Sequence 4: AN7354.1	131 aa
Alignment Length: 135 aa
Number Identitical Residues: 81 aa
Alignment Score (without introns) 3693


MG05248.1 	MVAAAKHTAI~VKKR1TKSFMRHQSDRFKRVDPSWRKPKGIDNRVRRRFRGNIAMPS0IG
NCU00464.1	MVAAKKHVPI~VKKR1SKGFMRHQSDRFMRVDSAWRKPKGIDNRVRRRFRGTLAMPS0IG
FG04137.1 	MVSAKKHVPI0R---~---FERHQHDRFMRVSPSWRKPKGIDSRVRRRFRGTIAMPS0IG
AN7354.1  	MVLAKKHVPI~VKKR1TKRFTRHQSDRFKCVPESWRKPKGIDNRVRRRFKSNIPMPS0IG
          	** * **..*  ..  :. * *** ***  *  :********.******:..:.*** **

MG05248.1 	YGSNKRTKHMMPSGHKAFLVQNINDVELLLMHNQVFAAD2SQSRIAHNVSSRKRIEIIAR
NCU00464.1	YGSNKKTRHMMPSGHKAFLVSNVKDVELLLMHNKTYAAE2----IAHNVSSRKRIEIIAR
FG04137.1 	YGSNKKTRYMAPSGFKAFIVSNKSDLELLLMHNKTHAAE2----IAHNVSSRKRVDIIAR
AN7354.1  	YGSNKKTKHMMPSGHKAFLVHNVKDVELLLMHNRTYAAE2----IASAVSSRKRVDIIAK
          	*****:*::* ***.***:* * .*:*******:..**:     **  ******::***:

MG05248.1 	AKQLGVKVTNPKAKVTTEV
NCU00464.1	AKQLSVKVTNAKAKVTTEV
FG04137.1 	AKQLGVKVTNPKAKVTTEV
AN7354.1  	AKALGVKVTNPRGRVTTEA
          	** *.*****.:.:****.
```
